# Perceptual–motor performance and neural conduction asymmetries in elite basketball players

**DOI:** 10.1186/s13102-026-01643-7

**Published:** 2026-03-12

**Authors:** Meltem Devrilmez, Recep Soslu, Erhan Devrilmez, Abdullah Uysal, Ömer Özer, Mustafa Şakir Akgül, Rohit Kumar Thapa, Ismail Can Çuvalcıoğlu, Hüseyin Şahin Uysal

**Affiliations:** 1 Karamanoğlu Mehmetbey University, Institute of Health Science, Karaman, 70200 Türkiye; 2 Department of Coaching Education, Karamanoğlu Mehmetbey University, Faculty of Sports Sciences, Karaman, 70200 Türkiye; 3 Faculty of Sport Science, Department of Physical Education and Sport, Bandırma Onyedi Eylül University, Balıkesir, 10010 Türkiye; 4 Department of Sport Management, Faculty of Sport Sciences, Kastamonu University, Kastamonu, 37150 Türkiye; 5 Symbiosis School of Sports Sciences, Symbiosis International (Deemed University), Pune, India; 6 Department of Coaching Education, Faculty of Sport Sciences, Burdur Mehmet Akif Ersoy University, Burdur, 15030 Türkiye

**Keywords:** Coincidence anticipation timing, Nerve conduction, Electromyography, Perceptual–motor performance, Hand dominance, Basketball

## Abstract

**Background:**

This study investigated whether the dominant hand would exhibit faster nerve conduction velocity, lower muscle activation latency, and superior Coincidence Anticipation Timing (CAT) performance compared to the non-dominant hand, reflecting enhanced neuromuscular efficiency and perceptual accuracy due to sport-specific training adaptations.

**Methods:**

This study employed a single-group, between subject comparative design to examine perceptual–motor performance and neuromuscular activation characteristics of the dominant and non-dominant hands. CAT and basal electromyographic (EMG) measurements were obtained separately for each hand, followed by task-related CAT performance during which EMG signals were recorded simultaneously. Comparisons between dominant and non-dominant hands were performed using independent samples t-tests.

**Results:**

The dominant hand exhibited significantly greater AE and VE across general and nerve-specific measures (*p* < 0.01), with large to very large effect sizes, indicating differences in perceptual timing accuracy and consistency. Median nerve latency was shorter in the dominant hand, whereas median nerve amplitude did not differ significantly between hands. In contrast, ulnar nerve latency was longer and ulnar nerve amplitude was lower in the dominant hand, reflecting asymmetrical peripheral neural characteristics. Task-induced changes analyses demonstrated significant increases in nerve latency and reductions in amplitude in both limbs (*p* < 0.001), suggesting neuromuscular strain following task execution. Correlation analyses revealed weak-to-moderate associations between CAT errors and EMG parameters (*r* = 0.25–0.45), indicating that perceptual–motor performance is only partially explained by peripheral neuromuscular activation.

**Conclusion:**

Elite basketball players demonstrate distinct perceptual–motor and neurophysiological asymmetries between dominant and non-dominant hands. The integration of CAT outcomes with sEMG and nerve conduction measures provides a comprehensive approach for characterizing perceptual–motor performance and neuromuscular function. These findings may inform bilateral training strategies and neuromuscular monitoring practices aimed at optimizing performance and reducing neural strain in basketball players.

**Trial registration:**

ClinicalTrials.gov NCT07253623, Date: 11.19.2025. Retrospectively registered.

## Introduction

In high-level athletic performance, effective execution depends on the close integration of perceptual–cognitive processing and neuromuscular coordination, particularly in open-skill sports such as basketball. These sports require athletes to continuously perceive, interpret, and respond to rapidly changing environmental stimuli under time pressure. One key perceptual–cognitive skill in this context is coincidence anticipation timing (CAT), defined as the ability to accurately predict both the temporal (“when”) and spatial (“where”) arrival of a moving stimulus [1]. CAT is considered a fundamental component of skilled performance in dynamic environments that demand rapid and precise responses to unpredictable events [2].

In basketball, players are constantly exposed to complex visual information, including ball trajectories, teammate movements, and opponent positioning, necessitating efficient visual search strategies and fast information processing. Accordingly, perceptual anticipation and decision-making speed are recognized as critical determinants of on-court performance [3]. CAT tasks provide an objective and standardized method for assessing spatial–temporal decision-making abilities across varying stimulus speeds [2]. Performance in these tasks is commonly quantified using absolute error (AE), which reflects overall temporal accuracy, and variable error (VE), which represents response consistency across trials [2].

Previous research has consistently shown that experienced basketball players outperform less experienced or novice individuals in CAT tasks, demonstrating greater timing accuracy and lower error values [4]. Expertise-related advantages have been attributed to more refined perceptual strategies and more efficient anticipation of task-relevant cues. In parallel, attentional focus has also been shown to influence motor performance, with an external focus of attention enhancing outcomes in basketball-specific skills such as free-throw shooting [5]. As expertise develops, athletes exhibit faster and more adaptable perceptual responses, facilitating smoother transitions to subsequent actions such as shooting, rebounding, or defensive movements [6].

However, perceptual–cognitive proficiency alone does not fully account for performance success. The execution of basketball-specific skills also relies heavily on neuromuscular efficiency, particularly the timely and coordinated activation of upper extremity muscles. Surface electromyography (sEMG) is widely used to assess neuromuscular characteristics such as muscle activation onset, amplitude, contraction velocity, and signal variability [7]. These parameters are known to vary depending on task demands, muscle group, and stimulus intensity [8]. In basketball contexts, training experience has been shown to influence upper-limb activation patterns, especially during precision tasks such as free throws [4]. Notably, greater variability in muscle activation timing has been observed during successful attempts, suggesting the use of flexible motor control strategies under competitive pressure [4]. Conversely, upper-limb fatigue has been associated with reductions in grip strength and passing accuracy, underscoring the functional relevance of neuromuscular readiness [9].

Despite substantial evidence supporting the independent roles of perceptual timing and neuromuscular activation in basketball performance, integrative research examining these domains concurrently remains limited. Most studies have addressed CAT performance or neuromuscular characteristics in isolation, restricting our understanding of how perceptual decision-making interacts with physiological readiness during performance [10]. In particular, the relationship between upper-limb neuromuscular conduction characteristics and timing accuracy has not been sufficiently explored. In perceptual–motor tasks performed under strict temporal constraints, performance is shaped by an inherent balance between response execution and temporal precision. Motor control research has consistently shown that enhanced neuromuscular efficiency and faster sensorimotor processing do not necessarily result in lower timing error, particularly when task demands emphasize rapid responses. In such contexts, skilled performers may exhibit increased absolute or variable error as a consequence of prioritizing efficient neural processing and rapid task execution. This principle suggests that higher error values may coexist with advanced neuromuscular function, especially in sport-specific tasks requiring rapid perceptual–motor integration.

Therefore, the primary aim of the present study was to investigate the relationship between CAT performance and neuromuscular activation characteristics of the dominant and non-dominant hands in female basketball players. Specifically, we examined whether neuromuscular parameters assessed via surface electromyography (sEMG), including muscle activation onset, contraction velocity, and signal variability, were associated with CAT performance indices, namely AE and VE. A secondary aim was to compare these relationships between the dominant and non-dominant hands to identify potential perceptual–motor asymmetries. Within the framework of the speed–accuracy trade-off, it was hypothesised that the dominant hand would demonstrate greater neuromuscular efficiency, reflected by faster neural conduction and shorter activation latency, while this enhanced efficiency under high temporal task demands could be accompanied by distinct patterns of CAT performance, including increased AE and VE, consistent with sport-specific training adaptations.

## Materials and methods

### Participants

This study employed a single-group, between-subjects comparative design to examine perceptual–motor performance and neuromuscular activation characteristics of the dominant and non-dominant hands. CAT and basal electromyographic (EMG) activity were obtained separately for each hand. Subsequently, CAT performance was assessed while EMG signals were recorded simultaneously during task execution. Fourteen elite female basketball players participated in the study. All athletes were actively competing in licensed basketball clubs within the Türkiye second second national league system and were engaged in regular team training (≥ 4 sessions/week). Competitive level and structured training history support the ecological validity of the sample. Prior to data collection, an a priori power analysis was conducted using G*Power software (version 3.1; [11]) for a repeated-measures design (dominant vs. non-dominant hand). Assuming a medium effect size (f = 0.25), an alpha level of 0.05, and statistical power of 0.80, the analysis indicated that a minimum of 12 participants was required. The final sample size of 14 participants therefore exceeded the minimum requirement. Participant characteristics were as follows (mean ± SD): age, 21.6 ± 1.4 years; height, 177.3 ± 4.9 cm; basketball experience, 9.3 ± 2.9 years; and body mass, 69.8 ± 11.0 kg. Inclusion criteria included no history of neurological disorders, no previous head injuries, and no upper-extremity injuries or surgeries within the previous six months. All participants were familiar with the testing procedures. The study protocol was approved by the Ethics Committee of Karamanoğlu Mehmetbey University, Faculty of Medicine (Approval No: 2023/45; Date: 12.09.2023) and was prospectively registered as a clinical trial at ClinicalTrials.gov (Identifier: NCT07253623; Date: 11/19/2025). All participants provided written informed consent prior to participation.

### Procedure

**Fig. 1:**
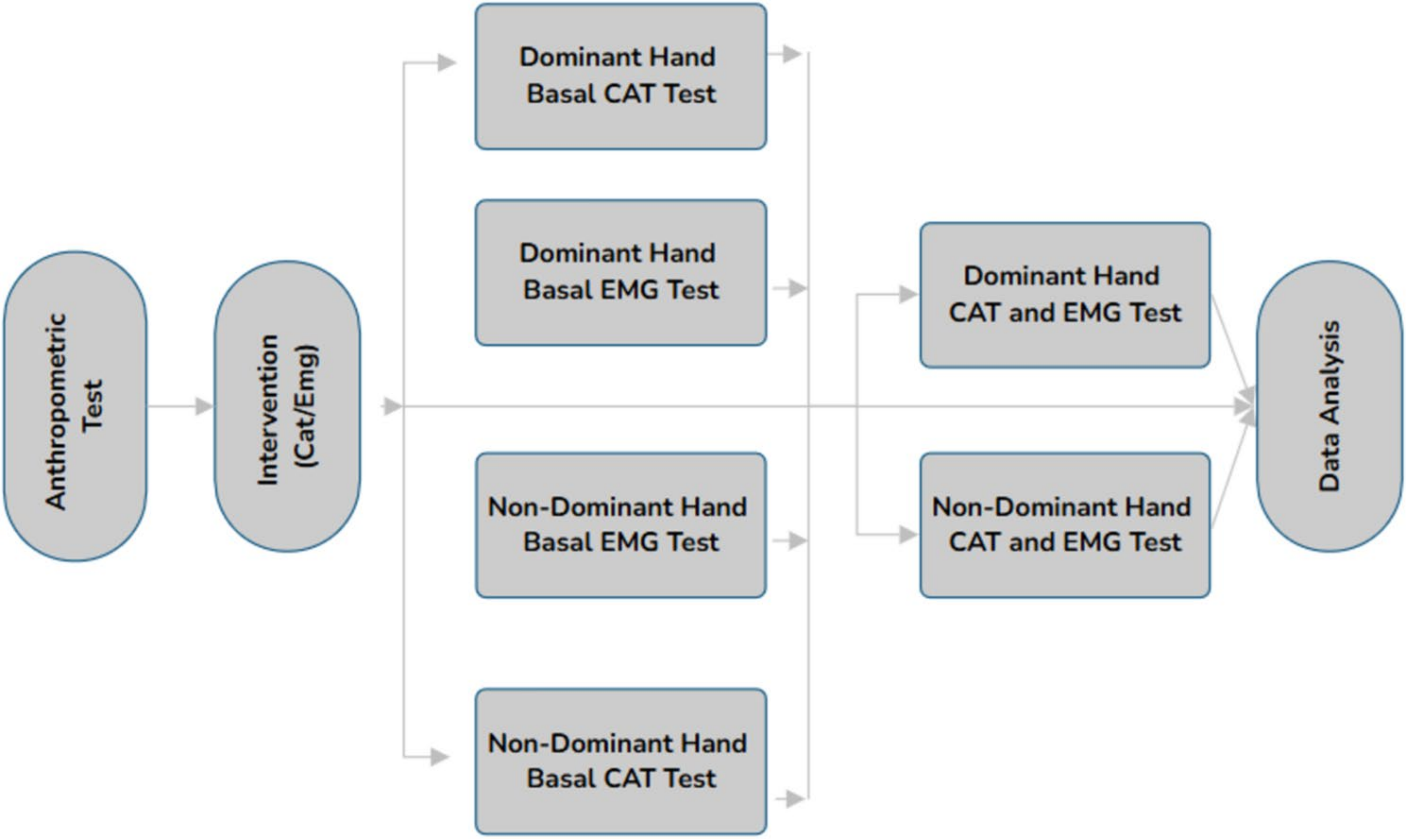
Experimental timeline of EMG acquisition and CAT testing

All testing procedures were conducted in a standardized and sequential manner. In the first stage, anthropometric assessments were performed, including height, body mass, and arm length. Hand dominance was determined based exclusively on sport-specific criteria, defined as the hand predominantly used for shooting in basketball, given that all participants were trained female basketball players. The contralateral arm was classified as the non-dominant limb. This sport-specific approach was adopted to ensure relevance to basketball performance and to avoid ambiguity associated with general daily activities. A separate familiarization session was not conducted, as all participants were experienced basketball players with prior exposure to anticipatory timing tasks and upper-extremity motor assessments as part of their regular training and testing routines. To minimize potential learning effects, all participants received standardized verbal instructions and practice trials immediately before data collection, which were considered sufficient to ensure task comprehension and reliable performance across sessions. All measurements were completed across three separate sessions conducted on non-consecutive days, with a minimum rest period of 48 h between sessions to prevent residual fatigue. Testing sessions were scheduled at the same time of day for each participant to control for circadian influences. All assessments were carried out in the same indoor laboratory environment under controlled conditions (ambient temperature: 22–24 °C, relative humidity: 45–55%, and standardized lighting). Participants were instructed to refrain from strenuous physical activity and caffeine consumption for 24 h prior to each session. Before each testing session, participants completed a standardized warm-up protocol lasting approximately 10 min, consisting of 5 min of light aerobic activity followed by dynamic upper-extremity movements (e.g., shoulder rotations, arm swings, and submaximal ball-handling drills). This warm-up was designed to increase muscle temperature, enhance neuromuscular readiness, and reduce injury risk while ensuring consistency across sessions. Following the warm-up, baseline measurements were obtained separately for the dominant and non-dominant hands. Participants performed the coincidence anticipation timing (CAT) task, during which temporal accuracy and response timing were recorded in response to standardized visual stimuli. During this phase, surface electromyography (sEMG) was used to assess neuromuscular activity of the biceps brachii and deltoid muscles under resting and preparatory conditions. In the experimental phase, participants performed the CAT task while sEMG signals were recorded simultaneously. The task was executed separately with the dominant and non-dominant hands to identify potential perceptual–motor and neuromuscular asymmetries. EMG electrodes remained fixed throughout the session to ensure consistent signal acquisition and precise synchronization between perceptual events and neuromuscular responses. Finally, CAT and EMG data were synchronized using time-locked event markers. CAT performance was quantified using AE and VE, while EMG signals were analyzed for muscle activation onset latency, signal amplitude, and intra-trial variability. These parameters were used to examine within-subject differences between dominant and non-dominant limbs and to evaluate perceptual–motor coordination patterns in trained basketball players (Fig. 1).

### Electromyography (EMG) recording and processing

Raw EMG signals were obtained using surface Ag/AgCl disc electrodes positioned according to the SENIAM guidelines, with an inter-electrode distance standardized at 2 cm. To minimize impedance, the skin was shaved, lightly abraded, and cleansed with alcohol before electrode placement. EMG data were acquired at a sampling rate of 1000 Hz using a Medelec/TECA Sapphire II 4E system (Oxford Instruments Medical, Surrey, United Kingdom). The signals were band-pass filtered between 20 and 450 Hz using a fourth-order Butterworth filter to suppress motion artifacts and high-frequency noise. A 50 Hz notch filter was additionally applied to remove power line interference. Processed signals were full-wave rectified and smoothed using a moving root mean square (RMS) window of 50 ms. Muscle activation onset was operationally defined as the point at which the rectified EMG amplitude exceeded three standard deviations above baseline noise for a minimum duration of 25 ms [12].

### Nerves medianus conduction velocity

For the median nerve motor conduction, Ag/AgCl surface electrodes were employed with the participant’s arm extended and palm turned upwards. The active electrode was placed on the index finger muscle (i.e., m. abductor pollicis brevis), whereas the reference electrode was situated on the thumb joint. The grounding electrode was placed between the wrist and the recording areas. Surface electrodes were positioned on the palm, at the distal wrist line, on the elbow, and in the axilla next to the brachial artery for stimulation [12].

### Nerves ulnaris conduction velocity

The ulnar nerve was selected for the stimulation and recording of compound nerve action potentials due to its superficial anatomical position, which allows for reliable electrode placement and efficient signal acquisition. To standardize the testing position, participants were positioned with the elbow flexed at an angle of 15°–30°, the forearm supinated, and the palm facing upward. The active (recording) electrode was placed over the abductor digiti minimi muscle, while the reference electrode was positioned on the associated muscular tendon approximately 3 cm distal to the active site. Electrical stimulation was applied at four specific anatomical points to assess segmental conduction: (1) 8 cm proximal to the active electrode at the wrist, (2) 2 cm distal to the medial epicondyle, (3) 3 cm proximal to the medial epicondyle, with a minimum inter-stimulation distance of 10 cm between the second and third sites, and (4) 4 cm inferior to the lower border of the brachial artery in the axilla. This protocol enabled the comprehensive evaluation of nerve conduction velocity and latency across multiple segments of the upper limb, providing a robust neurophysiological assessment framework [12].

### CAT measurement

The CAT was measured using a Bassin anticipation timer device. It features a 2.24-meter runway with 49 LED lights arranged in a linear pattern to simulate a moving stimulus. Participants start by observing a yellow warning light, followed by a sequence of red lights. Their task was to anticipate the arrival of the final target light and press a button when it lights up. The speed of the light sequence was set to 7 m/sec, based on findings from basketball free-throw studies that equate this speed to the average speed of a basketball [13]. The device was positioned one meter above the ground, and participants were seated at a distance of two meters from the middle of the runway. Warning light times varied randomly between 0.5 and 3.0 s to introduce variability. Each participant went through 3 practice trials followed by 20 testing trials—10 for each hand. The tests were conducted in a dark room to ensure focus and concentration, and each trial result was recorded in milliseconds to measure performance accurately.

### Data analysis

All statistical analyses were performed using appropriate parametric methods following verification of underlying assumptions. Data normality was assessed using the Shapiro–Wilk test, and homogeneity of variances was evaluated via Levene’s test. EMG and CAT tests were measured as pre- and post-tests. The difference between the tests was calculated for both EMG and CAT tests and reduced to a single measurement time. As the data met the assumptions for parametric testing, independent sample t-tests were used to compare dominant and non-dominant hands for AE, VE, and nerve conduction parameters. For AE and VE comparisons were conducted for general measures as well as median and ulnar nerve–specific variables. Similarly, median and ulnar nerve latency and amplitude values were compared between dominant and non-dominant hands using independent samples t-tests. Effect sizes (ES) were calculated using Cohen’s d, which is recommended for independent sample designs, and interpreted as small (0.2), moderate (0.5), and large (0.8). Results were presented as mean ± standard deviation (SD) along with 95% confidence intervals (CI). The level of statistical significance was set at *p* < 0.05 [14]. To examine associations between motor performance errors and neuromuscular activation, correlation analyses were performed separately for the dominant and non-dominant hands. Correlations between AE, VE, and EMG parameters were computed using Pearson’s correlation coefficient (r), as all variables demonstrated approximately normal distributions. Correlation strength was interpreted as weak (*r* < 0.30), moderate (*r* = 0.30–0.59), or strong (*r* ≥ 0.60). Correlation matrices were visualized using heatmap representations to illustrate the direction and magnitude of relationships among variables. All statistical analyses were performed using IBM SPSS Statistics for Windows, Version 24.0 (IBM Corp., Armonk, NY, USA).

## Results

Examination of Table 1 revealed that the overall absolute error results indicated that the dominant hand (DAE: 58.59 ± 31.67 m/sec) produced significantly greater absolute error than the non-dominant hand (NDAE: 50.28 ± 22.09 m/sec) (t = 6.136, *p* < 0.001, ES = 1.64). This effect size reflects a large dominance-related effect. A similar pattern was observed for median nerve absolute error. Absolute error values measured in the dominant hand (DMAE: 72.67 ± 21.28 m/sec) were significantly higher than those in the non-dominant hand (NDMAE: 58.32 ± 24.92 m/sec) (t = 7.763, *p* < 0.001, ES = 2.08). The magnitude of this effect indicates a very large difference attributable to hand dominance. Likewise, ulnar nerve absolute error values were significantly greater in the dominant hand (DUAE: 57.81 ± 22.35 m/sec) compared with the non-dominant hand (NDUAE: 51.80 ± 18.36 m/sec) (t = 8.579, *p* < 0.001, ES = 2.29). Notably, this comparison yielded the largest effect size among all analyzed parameters.

**Table 1:** Comparison of absolute error between dominant and non-dominant hands for general, median, and ulnar nerve measures

Variable	Mean ± SD	%95(CI)	t	*p*	*ES*
DAE (m/sec)	58.59 ± 31.67	(37.31–79.87)	6.136	0.001^***^	1.64
NDAE (m/sec)	50.28 ± 22.09	(35.44–65.12)			
DMAE (m/sec)	72.67 ± 21.28	(58.38–86.97)	7.763	0.001^***^	2.08
NDMAE (m/sec)	58.32 ± 24.92	(41.58–75.06)			
DUAE (m/sec)	57.81 ± 22.35	(42.79–72.82)	8.579	0.001^***^	2.29
NDUAE (m/sec)	51.80 ± 18.36	(36.46–64.14)			

Values are mean ± SD with 95% confidence intervals (CI)

*DAE *Dominant hand absolute error, *NDAE *Non-dominant hand absolute error, *DMAE *Dominant hand median nerve absolute error, *NDMAE *Non-dominant hand median nerve absolute error, *DUAE *Dominant hand ulnar nerve absolute error, *NDUAE *Non-dominant hand ulnar nerve absolute error, *ES *Effect size

***p < 0.001

Results presented in Table 2 indicate significant differences in variable error (VE) between the dominant and non-dominant hands across general and nerve-specific measures. For overall variable error, the dominant hand (DVE: 41.81 ± 58.69 m/sec) exhibited significantly higher variability than the non-dominant hand (NDVE: 40.17 ± 43.25 m/sec) (t = 3.081, *p* < 0.05, ES = 0.82), corresponding to a large effect size. With respect to the median nerve, variable error values were markedly greater in the dominant hand (DMVE: 67.64 ± 20.87 m/sec) compared with the non-dominant hand (NDMVE) (t = 8.751, *p* < 0.001, ES = 2.87), indicating a very large dominance-related effect. Similarly, ulnar nerve variable error differed significantly between hands, with the dominant hand (DUVE: 46.51 ± 47.19 m/sec) showing greater variability than the non-dominant hand (NDUVE: 50.26 ± 15.73 m/sec) (t = 7.600, *p* < 0.01, ES = 2.83). This comparison also reflects a very large effect size.

**Table 2:** Comparison of variable error between dominant and non-dominant hands for general, median, and ulnar nerve measures

Variable	Mean ± SD	%95(CI)	t	*p*	*ES*
DVE (m/sec)	41.81 ± 58.69	(23.79–81.24)	3.081	0.05^*^	0.82
NDVE (m/sec)	40.17 ± 43.25	(11.12–69.22)			
DMVE (m/sec)	67.64 ± 20.87	(53.62–81.66)	8.751	0.001^***^	2.87
NDMVE (m/sec)	33.34 ± 57.77	(54.96–72.15)			
DUVE (m/sec)	46.51 ± 47.19	(14.81–78.21)	7.600	0.01^**^	2.83
NDUVE (m/sec)	50.26 ± 15.73	(39.70-60.83)			

Values are mean±SD with 95% confidence intervals (CI)

*DVE *Dominant hand variable error, *NDVE *Non-dominant hand variable error, *DMVE *Dominant hand median nerve variable error, *NDMVE* Non-dominant hand median nerve variable error, *DUVE *Dominant hand ulnar nerve variable error, *NDUVE* Non-dominant hand ulnar nerve variable error, *ES *Effect size

*p<0.05

**p<0.01

***p<0.001

Results presented in Table 3 demonstrate differences in median nerve latency and amplitude between the dominant and non-dominant hands. Median nerve latency was significantly shorter in the dominant hand (DML:6.50 ± 0.36 ms; 95% CI: 6.29–6.71) compared with the non-dominant hand (NDML: 6.62 ± 0.36 ms; 95% CI: 6.41–6.83) (t = − 5.896, *p* < 0.001, ES = 1.58). The magnitude of the effect size indicates a large dominance-related difference in median nerve conduction latency. In contrast, median nerve amplitude did not differ significantly between hands. Although the dominant hand exhibited slightly higher amplitude values (DMA: 7.03 ± 2.31 ms; 95% CI: 5.70–8.36) compared with the non-dominant hand (NDMA: 6.86 ± 1.60 ms; 95% CI: 5.94–7.78), this difference was not statistically significant (t = − 1.152, *p* > 0.27, ES = 0.31), corresponding to a small effect size.

**Table 3:** Comparison of median nerve latency and amplitude between dominant and non-dominant hands

Variable	Mean ± SD	%95(CI)	t	*p*	*ES*
DML (ms)	6.50 ± 0.36	(6.29–6.71)	-5.896	0.001***	1.58
NDML (ms)	6.62 ± 0.36	(6.41–6.83)			
DMA (ms)	7.03 ± 2.31	(5.70–8.36)	-1.152	0.27	0.31
NDMA (ms)	6.86 ± 1.60	(5.94–7.78)			

Values are mean±SD with 95% CI

*DML* Dominant hand median nerve latenc, *NDML* Non-dominant hand median nerve latency, *DMA* Dominant hand median nerve amplitude, *NDMA* Non-dominant hand median nerve amplitude, *ES* Effect size

***p<0.001

Results presented in Table 4 reveal significant differences in ulnar nerve latency and amplitude between the dominant and non-dominant hands. Ulnar nerve latency was significantly longer in the dominant hand (DUL: 6.56 ± 0.50 ms; 95% CI: 6.27–6.85) compared with the non-dominant hand (NDUL: 6.19 ± 0.37 ms; 95% CI: 5.98–6.40) (t = − 4.288, *p* < 0.001, ES = 1.15). The magnitude of the effect size indicates a large dominance-related difference in ulnar nerve conduction latency. In addition, ulnar nerve amplitude differed significantly between hands. The dominant hand exhibited lower amplitude values (DUA: 10.05 ± 1.65 ms; 95% CI: 9.10–11.00) compared with the non-dominant hand (NDUA: 11.67 ± 2.15 ms; 95% CI: 10.43–12.91) (t = − 2.370, *p* < 0.03, ES = 0.63), corresponding to a moderate effect size.

**Table 4:** Comparison of ulnar nerve latency and amplitude between dominant and non-dominant hands

Variable	Mean ± SD	%95(CI)	t	*p*	*ES*
DUL (ms)	6.56 ± 0.50	(6.27–6.85)	-4.288	0.001***	1.15
NDUL (ms)	6.19 ± 0.37	(5.98–6.40)			
DUA (ms)	10.05 ± 1.65	(9.10–11.00)	-2.37	0.03*	0.63
NDUA (ms)	11.67 ± 2.15	(10.43–12.91)			

Values are mean±SD with 95% confidence intervals (CI)

*DUL *Dominant hand ulnar nerve latency, *NDUL *Non-dominant hand ulnar nerve latency, *DUA *Dominant hand ulnar nerve amplitude, *NDUA *Non-dominant hand ulnar nerve amplitude, *ES *Effect size

*p<0.05

***p<0.001

**Fig. 2:**
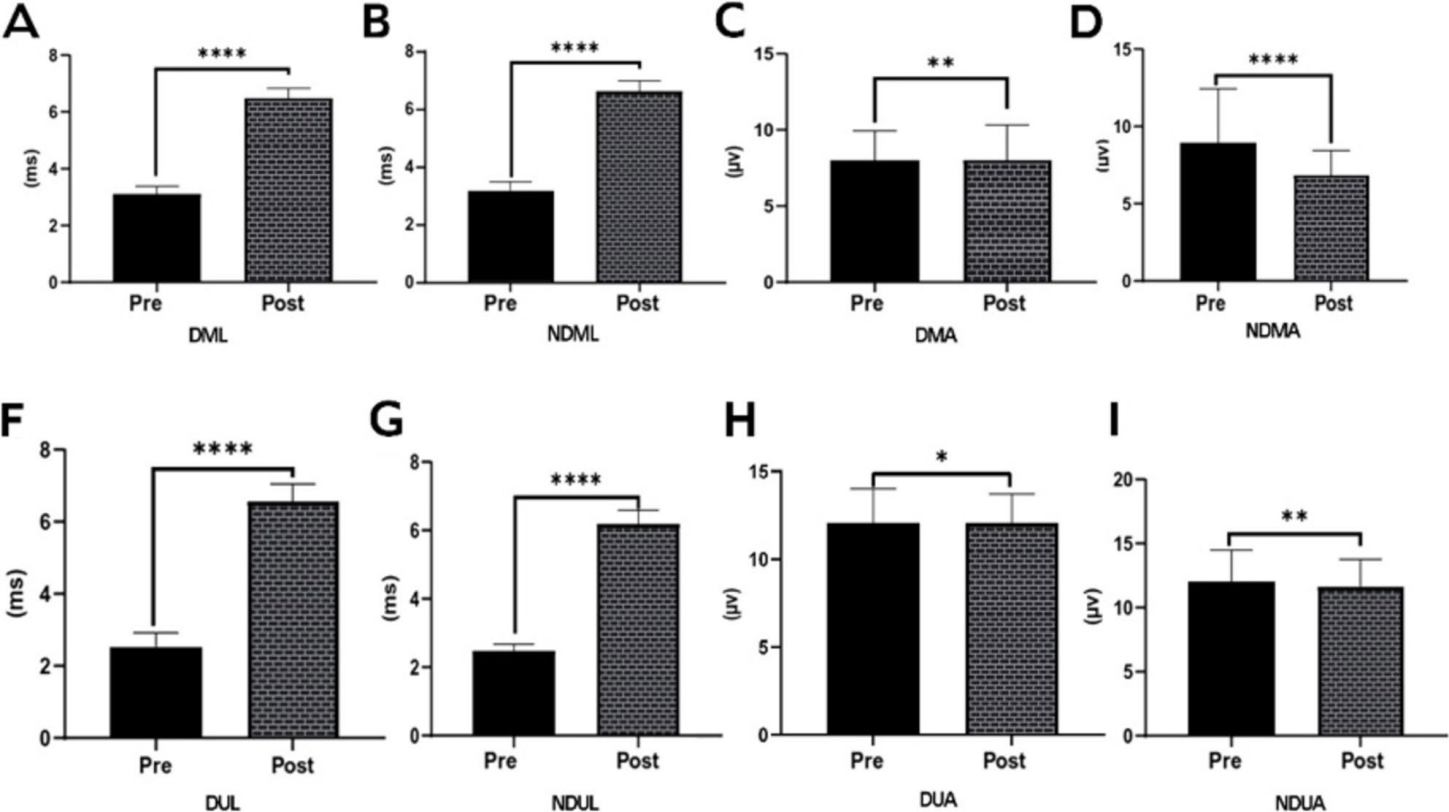
Pre–post changes in median and ulnar nerve latency and amplitude parameters. Panels **A** and **B** show median nerve latency for the dominant (DML) and non-dominant (NDML) hands, respectively. Panels **C** and **D** illustrate median nerve amplitude for the dominant (DMA) and non-dominant (NDMA) hands. Panels **F** and **G** present ulnar nerve latency for the dominant (DUL) and non-dominant (NDUL) hands, while panels **H** and **I** depict ulnar nerve amplitude for the dominant (DUA) and non-dominant (NDUA) hands. Data are presented as mean ± SD. Statistical significance for pre–post comparisons is indicated by asterisks (*p < 0.05, **p < 0.01, ****p < 0.001)

Figure 2 illustrates task-induced changes in median and ulnar nerve latency and amplitude parameters. Median nerve latency increased following task execution in both the dominant and non-dominant hands (DML; panel A) and non-dominant hands (NDML; panel B) (*p* < 0.001 for both). Median nerve amplitude showed a modest but significant reduction in the dominant hand (DMA; panel C, *p* < 0.01), whereas a more pronounced decrease was observed in the non-dominant hand (NDMA; panel D, *p* < 0.001). Similarly, ulnar nerve latency increased significantly following the intervention in both the dominant (DUL; panel F) and non-dominant hands (NDUL; panel G) (*p* < 0.001 for both). With respect to ulnar nerve amplitude, a small but significant reduction was detected in the dominant hand (DUA; panel H, *p* < 0.05), while a moderate decrease was observed in the non-dominant hand (NDUA; panel I, *p* < 0.01). Figure 2 demonstrates a consistent following task execution prolongation of nerve conduction latency accompanied by reductions in nerve amplitude, with larger and more consistent effects observed for latency-related parameters across both hands.

**Fig. 3:**
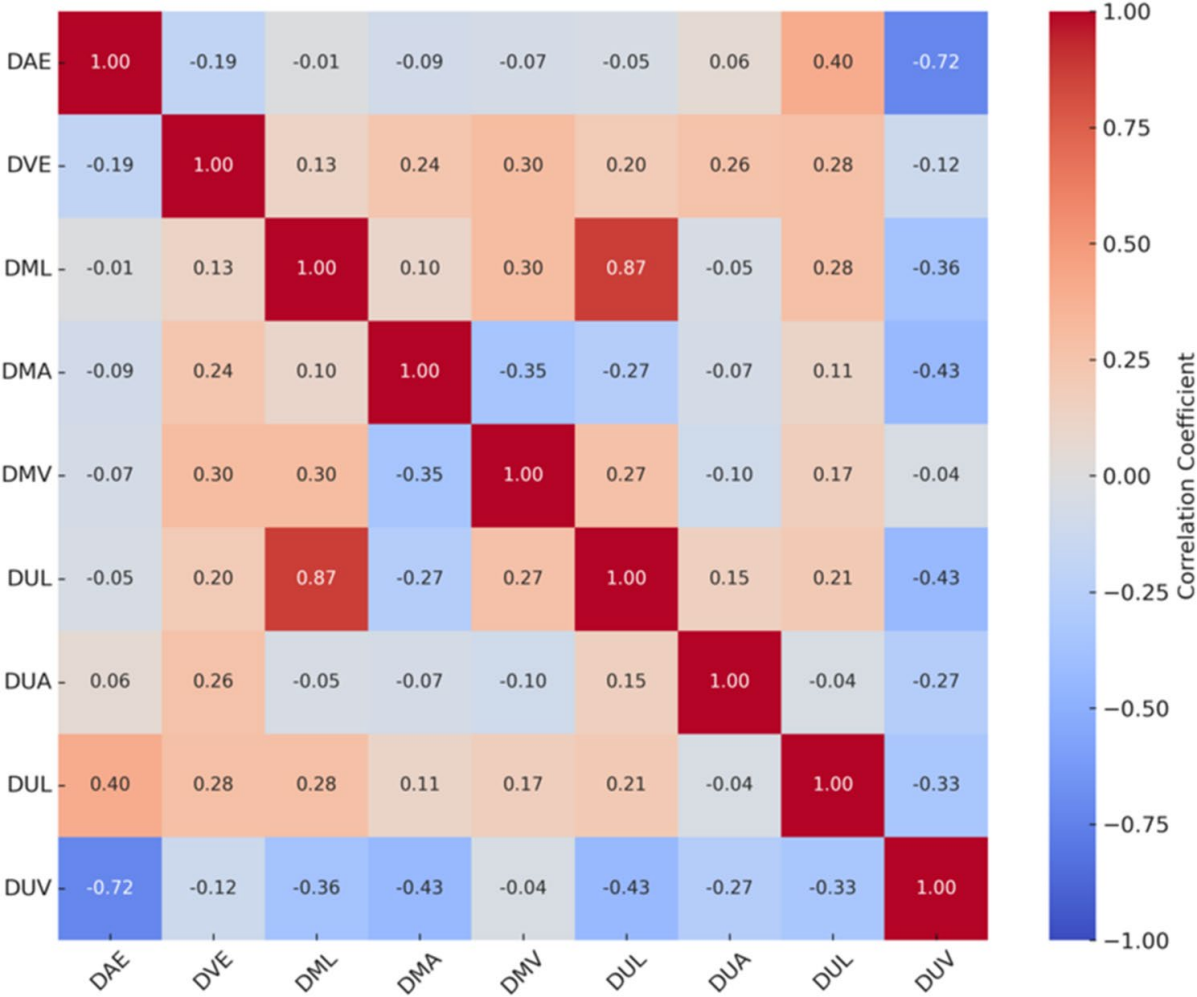
Correlation matrix between AE, VE, and EMG parameters of the dominant hand

Figure 3 illustrates the correlations between AE, VE, and EMG parameters of the dominant hand. AE measures were predominantly negatively associated with EMG variables, with correlation coefficients typically ranging between (*r* = − 0.30 and − 0.45), indicating that higher AE values tended to coincide with lower levels of neuromuscular activation. VE parameters displayed both positive and negative associations with EMG measures, generally of small-to-moderate magnitude (*r* = − 0.25 to 0.35), depending on the specific variable examined. In contrast, EMG variables showed moderate positive intercorrelations (*r* = 0.40–0.60), reflecting consistent relationships among muscle activation indices. These findings indicate that dominant-hand performance errors are related to neuromuscular activation patterns, although the strength of these associations remains limited, suggesting that additional central or coordinative mechanisms contribute to AE and VE outcomes.

**Fig. 4:**
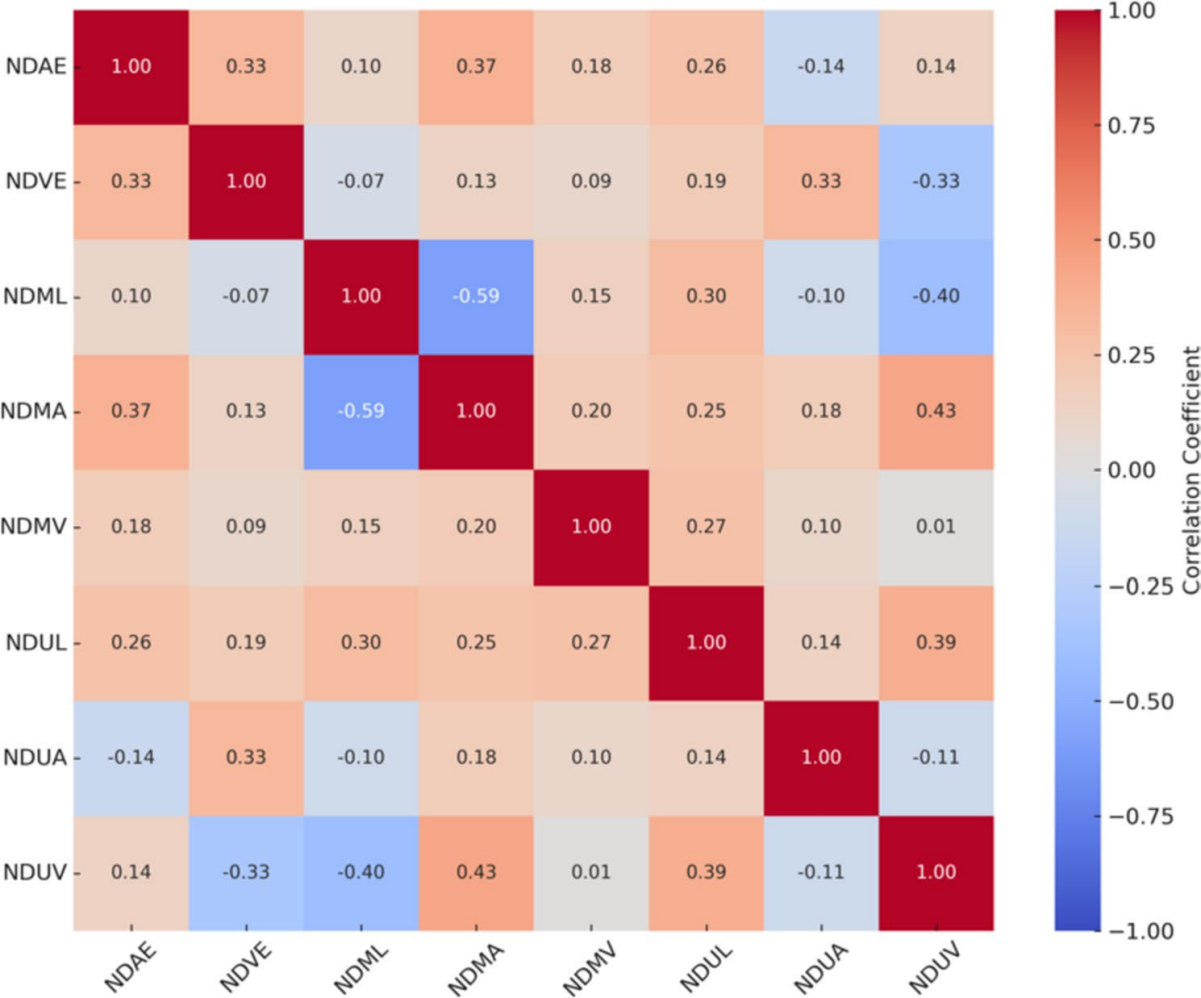
Correlation matrix between AE, VE, and EMG parameters of the non-dominant hand

Figure 4 depicts the correlations between AE, VE, and EMG parameters of the non-dominant hand. AE measures showed primarily weak-to-moderate negative correlations with EMG variables, with coefficients generally ranging from approximately (*r* = − 0.25 to − 0.45), indicating that increased AE tended to be associated with reduced neuromuscular activation. VE parameters demonstrated mixed associations with EMG measures, displaying both positive and negative correlations of small-to-moderate magnitude (*r* = − 0.20 to 0.40), depending on the specific EMG variable. EMG parameters themselves exhibited moderate positive intercorrelations (*r* = 0.40–0.60), suggesting consistent relationships among muscle activation indices. These findings indicate that, similar to the dominant hand, non-dominant hand performance errors are related to EMG activity; however, the modest strength of these correlations suggests that error-related performance outcomes are influenced by factors beyond peripheral neuromuscular activation alone.

## Discussion

The present study provides evidence that perceptual–motor performance in basketball is shaped by asymmetric neural adaptations favoring the dominant hand. In this regard, the dominant hand demonstrated distinct response characteristics during coincidence anticipation tasks, accompanied by greater absolute and variable temporal error. In contrast, the non-dominant hand showed more stable timing performance alongside comparatively slower neural conduction. Taken together, these findings indicate that perceptual–motor performance reflects differential neural organization between limbs rather than uniform optimization across the upper extremities.

To better contextualize these observations, it is important to consider the specific perceptual–motor demands of basketball. Basketball represents a highly dynamic environment requiring continuous extraction of visual information, prediction of opponents’ actions, and rapid execution of upper-limb movements under temporal constraints. Previous studies have shown that skilled basketball players display superior anticipatory abilities during interceptive actions, largely attributable to enhanced use of kinematic cues derived from body movements and ball trajectories [14]. In parallel, neurophysiological evidence suggests that a substantial proportion of performance-relevant information in basketball is processed through visual pathways [15, 16], emphasizing the central role of efficient visual–motor integration. Within this framework, coincidence anticipation timing tasks serve as a valid behavioral indicator of how perceptual input is translated into temporally precise motor responses [2, 17].

The observation of greater absolute and variable error in the dominant hand should be interpreted as a functional adaptation rather than a performance limitation. Long-term basketball training places repeated demands on the dominant limb for rapid perceptual–motor integration and task execution under high temporal constraints. Under such conditions, enhanced responsiveness may be accompanied by modest increases in temporal variability, reflecting adaptive adjustments in motor control strategies rather than diminished perceptual or motor capacity. This pattern therefore represents a neuromuscular profile shaped by sport-specific experience. Consistent with this interpretation, findings from the present study align with previous research conducted in basketball and other interceptive sports, supporting the presence of shared underlying mechanisms related to perceptual–motor expertise. Studies reporting superior coincidence anticipation timing performance in skilled athletes suggest that long-term training enhances the coupling between visual perception and motor planning, likely through reinforced afferent–efferent signaling and improved cortical–subcortical integration [18–21]. In the current study, dominance-related differences in neural conduction parameters may reflect reinforced corticospinal pathways and peripheral nerve adaptations arising from repetitive shooting, passing, and ball-handling actions.

Dominance-related adaptations should not be interpreted independently of sport-specific movement patterns and loading characteristics. Comparisons across sports indicate that volleyball and archery impose highly unilateral and repetitive upper-limb demands, often resulting in pronounced dominance-related differences in nerve conduction velocity [22, 23]. Basketball, by contrast, combines unilateral precision tasks such as shooting with substantial bilateral coordination during catching, rebounding, and defensive actions. This mixed demand structure may explain why dominance-related effects in basketball players appear evident yet not extreme, suggesting a balance between specialization and bilateral functional engagement [24]. Neuroimaging evidence further supports this interpretation by demonstrating increased activation in regions such as the inferior parietal lobe and frontal gyrus during action anticipation in experienced basketball players [19]. These regions are critically involved in integrating visual information with motor intentions and updating predictive models of action. Accordingly, shorter response latencies observed in the dominant hand may reflect enhanced central processing efficiency in addition to peripheral neural adaptations.

Consideration of peripheral neural mechanisms provides additional insight into the observed findings. At the peripheral level, exercise-induced changes in nerve conduction velocity have been linked to improved myelination, increased axonal diameter, and enhanced neuromuscular transmission efficiency [22, 25]. At the same time, the literature indicates that excessive or repetitive upper-limb loading can lead to subclinical neural stress in certain sporting contexts [26]. In basketball, upper-limb loading is frequent yet distributed across both limbs, a factor that may favor predominantly facilitative neural adaptations rather than degenerative changes. Following task execution, the findings revealed a partial reduction in functional and neural disparities between the dominant and non-dominant hands, suggesting that repeated engagement in sport-specific tasks may promote bilateral neural adaptation over time. This effect may be particularly relevant for the non-dominant limb, which plays a critical role in stabilizing and coordinating complex movements. Associations between coincidence anticipation timing performance and nerve conduction parameters further indicate that perceptual anticipation and peripheral neural efficiency represent interconnected components of perceptual–motor expertise. From an applied perspective, these findings emphasize the importance of integrating perceptual–motor training with neuromuscular monitoring in basketball, as bilateral training approaches may enhance anticipatory performance while supporting balanced neural adaptation and informing load management and injury prevention strategies.

### Limitations

Despite its contributions, this study has several limitations that should be considered when interpreting the findings. First, the use of linear and unidirectional visual stimuli in the coincidence anticipation timing (CAT) tasks may limit ecological validity, as real-game basketball environments involve multidirectional, unpredictable, and opponent-driven movement patterns. Therefore, the observed responses should be interpreted as task-specific perceptual–motor behavior rather than direct representations of in-game performance. Second, the study employed a single-group, within-subject comparative design without a control group, which restricts the ability to draw causal conclusions. Consequently, the observed task-related changes may reflect acute responses, such as fatigue, task repetition, or familiarization effects, rather than definitive neural or perceptual adaptation. Third, the sample consisted solely of female basketball players with varying levels of playing experience, which may have introduced heterogeneity and limited internal validity. Additionally, the relatively small sample size (*n* = 14) reduces statistical power, limits generalizability, and increases the risk of Type II error. Future research should aim to include larger and more homogeneous samples, incorporate male athletes for comparative purposes, and employ more ecologically valid, game-like tasks. Moreover, the integration of central nervous system measures (e.g., EEG, fMRI, TMS) and longitudinal training designs with appropriate control conditions may provide deeper insight into the neural and perceptual–motor processes underlying performance in basketball.

## Conclusions

This study demonstrates that asymptomatic basketball players exhibit distinct neurophysiological characteristics in the dominant limb, reflected by differences in median and ulnar nerve conduction parameters. These findings indicate that long-term, sport-specific training is associated with functional neural adaptations in the upper extremity. Repetitive upper-limb loading inherent to basketball-specific movements may also be related to neuromuscular strain, suggesting that observed neurophysiological alterations require cautious interpretation. Greater absolute and variable error in CAT performance, particularly in the dominant hand, indicates that perceptual–motor accuracy is only partially explained by peripheral neural efficiency alone. CAT outcomes reflect combined influences of neuromuscular activation patterns and task-specific perceptual–motor demands. Weak-to-moderate associations between CAT errors and electromyographic parameters support the contribution of central control mechanisms to timing performance. These findings highlight the complex interplay between neurophysiological adaptations and perceptual–motor performance in basketball players. From an applied perspective, the results emphasize the importance of balanced training and recovery strategies that support sport-specific neuromuscular adaptations while monitoring neural load to reduce the risk of cumulative neuromuscular strain.

## Data Availability

Data can be available upon reasonable request to the corresponding author.

## Abbreviations

DAE: Dominant hand Absolute Error
NDAE: Non-dominant hand Absolute Error
DMAE: Dominant hand N.Medianus Absolute Error
NDMAE: Non-dominant hand N.Medianus Absolute Error
DUAE: Dominant hand N.Ulnaris Absolute Error
NDUAE: Non-dominant hand N.Ulnaris Absolute Error
DVE: Dominant hand Variable Error
NDVE: Non-dominant hand Variable Error
DMVE: Dominant hand N.Medianus Variable Error
NDMVE: Non-dominant hand N.Medianus Variable Error
DUVE: Dominant hand N.Ulnaris Variable Error
NDUVE: Non-dominant hand N.Ulnaris Variable Error
DML: Dominant hand N.Medianus Latency (ms)
NDML: Non-dominant hand N.Medianus Latency (ms)
DMA: Dominant hand N.Medianus Amplitude (µV)
NDMA: Non-dominant hand N.Medianus Amplitude (µV)
DUL: Dominant hand N.Ulnaris Latency (ms)
NDUL: Non-dominant hand N.Ulnaris Latency (ms)
DUA: Dominant hand N.Ulnaris Amplitude (µV)
NDUA: Non-dominant hand N.Ulnaris Amplitude (µV)
